# A Micro-Resonant Gas Sensor with Nanometer Clearance between the Pole Plates

**DOI:** 10.3390/s18020362

**Published:** 2018-01-26

**Authors:** Xiaorui Fu, Lizhong Xu

**Affiliations:** Mechanical Engineering Institute, Yanshan University, Qinhuangdao 066004, China; fxr@stumail.ysu.edu.cn

**Keywords:** nanometer clearance, micro-resonant sensor, gas sensor

## Abstract

In micro-resonant gas sensors, the capacitive detection is widely used because of its simple structure. However, its shortcoming is a weak signal output caused by a small capacitance change. Here, we reduced the initial clearance between the pole plates to the nanometer level, and increased the capacitance between the pole plates and its change during resonator vibration. We propose a fabricating process of the micro-resonant gas sensor by which the initial clearance between the pole plates is reduced to the nanometer level and a micro-resonant gas sensor with 200 nm initial clearance is fabricated. With this sensor, the resonant frequency shifts were measured when they were exposed to several different vapors, and high detection accuracies were obtained. The detection accuracy with respect to ethanol vapor was 0.4 ppm per Hz shift, and the detection accuracy with respect to hydrogen and ammonias vapors was 3 ppm and 0.5 ppm per Hz shift, respectively.

## 1. Introduction

Micro-sensors have a compact structure, a low cost, low power loss, a high response speed, high accuracy, etc. [1,2]. The micro-resonant sensor outputs frequency signals and is suitable for the distant range transmission of certain signals such as measurements of the chemical constituents and pressure inside the gut [3,4,5]. The micro-resonant gas sensor is one type of micro-resonant sensor and is used to detect dangerous and harmful gas [6,7,8]. In a micro-resonant gas sensor, the measurement of the resonator vibration displacements is necessary. Presently, there are three main types of methods of measuring resonator vibration displacements.

The first is the piezoresistive method. Here, piezoresistive elements are integrated into the resonator during fabrication. Resonator bending displacement is proportional to the change in resistance. The change in resistance is measured with a Wheatstone bridge at the resonator root. With this method, octane, carbon monoxide, and several volatile organic gases have been detected [9,10,11]. Accuracy of the detection of alcohol can reach 14 ppm [12]. Moreover, a self-actuating and detecting loop has been fabricated, and experiments on the mechanical resonance of the micro-beam and the resonance of the electric signal have been discussed and analyzed [13]. Furthermore, a piezoresistive silica-type microcantilever resonator with a Cu^2+^/mercapto-undecanoic acid (11-MUA) self-assembled layer has been designed, and detection of methyl phosphonic acid dimethyl ester (DMMP) gas was achieved [14,15]. Piezoresistive detection has been widely used to measure resonator vibration displacements, but its drawbacks include thermal drifts, poor sensitivity, and the fact that the sensors are difficult to fabricate because the piezoresistive elements must be integrated into the micro-resonator.

The second is the optical method, which involves the reflection of a beam of light off the resonator onto a segmented photodiode or a position-sensitive detector [16]. A small mirror is attached to a cantilever, and the position of the laser beam that bounces off this mirror can then be monitored using a position sensitive photo-detector, which can discern 10–14 m changes in the cantilever bending [17]. The optical detection technique is the most sensitive method for measuring resonator vibration displacements. The drawback of the method is that their dimensions are large, and the detection system is expensive.

The third is the capacitance method. This method is based on measuring the capacitance between a conductor on the resonator and another fixed conductor on the substrate that is separated from the resonator by a small gap. Changes in the gap due to resonator displacement result in changes in the capacitance between two conductor plates. A resonant gas sensor for the detection of low-power capacitors with a PDMS (polydimethylsiloxane) as a sensitive layer with a thickness of 2.2 μm was developed and mainly used for the detection of toluene and octane. A low-power-loss resonant gas sensor by capacity measuring was produced, and the accuracy of the detection of toluene could reach 50 ppm [18]. A mechanical chemical coupled dynamics equation for a micro-resonant gas sensor was proposed, and the time frequency property of the resonant cantilever during gas adsorption reaction was investigated [19]. The capacitive method is widely used because of its simple structure requirements [20]. However, its shortcoming is weak signals due to small capacitance changes during resonator vibration. In efforts to overcome this weakness, improvement in the measuring circuit by increasing the amplifying factor and decreasing noise has been an area of focus.

Since the capacitance of a flat capacitor is inversely proportional to the separation distance, the sensitivity of this method relies on a very small gap between the resonator and the substrate. Hence, we propose a novel idea for increasing the signals of the capacitance change. We want to reduce the initial gap between the pole plates to the nanometer level, so that the capacitance between the pole plates and its change during resonator vibration can be increased significantly.

In this paper, a fabricating process of the micro-resonant gas sensor is proposed to deduce the initial clearance between pole plates at the nanometer level. A micro-resonant gas sensor with a 200 nm initial clearance was fabricated. Its measuring system was designed and fabricated with one-port electrostatic excitation and capacitive detection. The sensor’s sensitivity to ethanol vapor was tested, and the resonant frequency shift measured, when exposed to ethanol vapor. Results show that the detection accuracy with respect to ethanol vapor using the sensor is about 0.4 ppm per Hz shift.

## 2. Fabrication

**Figure 1:**
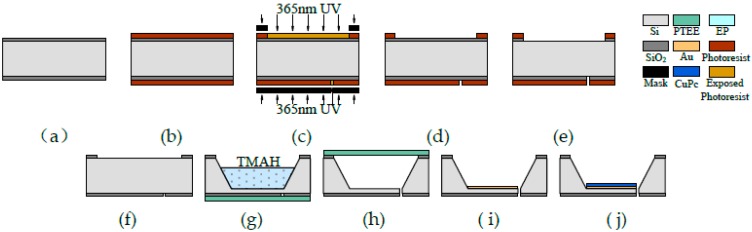
Fabrication process of the first silicon wafer. (**a**–**j**) Steps 1–10.

**Figure 2:**
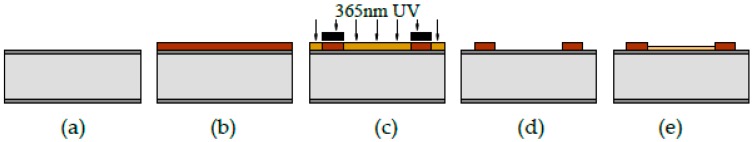
Fabrication process of the second silicon wafer. (**a**–**e**) Steps 1–5.

**Figure 3:**
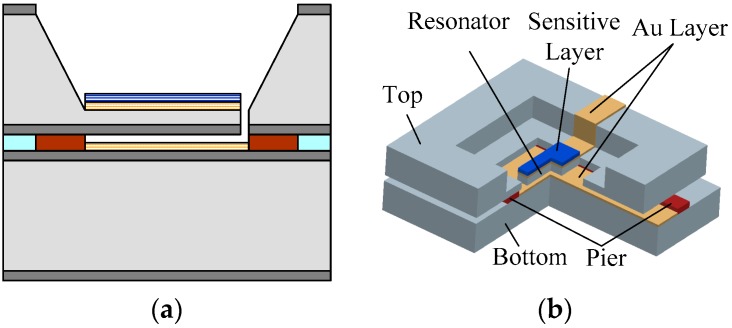
Two silicon wafers bonded. (**a**) Schematic diagram of cross section; (**b**) 3D diagram.

Here, a micro-resonant gas sensor with nanometer clearance was fabricated. The fabricating process of the micro-resonant gas sensor is shown in Figure 1, Figure 2 and Figure 3. The starting materials were 2 in. <100> 370 μm silicon wafers with double-sided thin silicon oxide 10,000 ± 300 Å in thickness. Two silicon wafers were used. 

The fabrication process for the first silicon wafer involves ten steps:

The first step is to remove organic matter from the silicon wafer with acetone and then to clean and dry it (see Figure 1a). The second step is to coat the silicon wafer with a positive photoresist 1 μm in thickness (see Figure 1b). The third step is to expose the silicon wafer to ultraviolet light (see Figure 1c). The fourth step is to develop the ultraviolet light on the silicon wafer with a 2.38% TMAH (tetramethylammoniumhydroxide) developer for 30 s (see Figure 1d). The fifth step is to remove silicon oxide from the silicon wafer in an HF (Hydrofluoric acid) solution for 30 min (see Figure 1e). The sixth step is to remove the photoresist from the silicon wafers in a 5% NaOH solution (see Figure 1g). The seventh step is to etch Si with a depth of about 350 μm using Teflon as the mask on the back side (see Figure 1h). The eighth step is to etch Si on the back side and to go through it to obtain a cantilever using Teflon as the mask on the front side (see Figure 1i). The ninth step is to, by means of lift-off technology, deposit Au onto the back side of the cantilever and deposit sensitive material phthalocyanine copper onto the Au film (see Figure 1j). The phthalocyanine copper layer is sensitive to ethanol vapor. Phthalocyanine copper has been reported to be able to detect several types of gases such as alcohol, NH_3_, and NO_2_. because the current–voltage characteristics on the phthalocyanine copper layer can be changed when these gases adsorb to the phthalocyanine copper layer [21,22,23]. Here, we exploit the adsorption ability of the phthalocyanine copper layer, but we did not detect changes in current–voltage characteristics. By detecting the natural frequency shift (mass changes of the micro-resonator), we measured the density of these gases. 

The fabrication process for the second silicon wafer involves five steps:

The first step is to remove organic matter from the silicon wafer and then to clean and dry it (see Figure 2a). The second step is to coat one side of the silicon wafer with a positive photoresist 1 μm in thickness (see Figure 2b). The third step is to expose the silicon wafer to ultraviolet light (see Figure 2c). The fourth step is to develop the ultraviolet light on the silicon wafer and then dry the two remaining photoresists, which will be taken as bearings (see Figure 2d). The fifth step is to deposit an 800 nm layer of Au between the two bearing photoresists by means of lift-off technology (see Figure 2e). 

Finally, two silicon wafers were bonded together with epoxy resin to obtain the micro-gas sensor with a 200 nm clearance between the cantilever and the base plate (see Figure 3). The 200 nm clearance is the gap between the top wafer and the bottom wafer with a 200-nm-thick step formed by the 1-μm-thick photoresists and the 800-nm-thick Au layer.

## 3. Measuring System

Here, one-port electrostatic excitation and capacitive detection was used. The resonator consisted in both an exciting unit and a detection unit. The exciting signal is

(1) u(t)=usinωt

where *u*(*t*) is the voltage of the exciting signal, *u* is its amplitude; *t* is the time; *ω* is the frequency of the exciting signal.

Under the above excitation, the electrostatic force between the resonant beam and the base plate is

(2) $$F_{e}=-\frac{1}{2}u^{2}\frac{dC}{d\omega }=-\frac{1}{2}u^{2}\sin^{2}(\omega t)\frac{dC}{d\omega }=\frac{\cos(2\omega t)-1}{4}u^{2}\frac{dC}{d\omega }.$$

Equation (2) shows that the frequency of the exciting force is 2*ω* when the frequency of the exciting signal is *ω*. Thus, the vibrating displacement of the resonant beam is

(3) y=Asin(2ωt+φ)

where *A* is the vibrating amplitude of the resonant beam, and f is its initial phase.

The capacitance between the resonator and the base plate is

(4) $$C=\frac{\varepsilon_{0}\varepsilon bl}{d-A\sin(2\omega t+\varphi )}.$$

The pole plate current is

(5) $$i=\frac{d(Cu)}{dt}=C\cdot A\cdot u\cdot \omega \cdot \cos(\omega t)+\frac{C^{2}\cdot A\cdot u\cdot \omega }{\varepsilon_{0}\varepsilon bl}\cdot (\sin(3\omega t+\varphi )-\sin(\omega t+\varphi )).$$

In Equation (5), there are signals of the frequencies *ω* and 3ω. The 3ω signal can be expressed as

(6) $$i{\;}_{3\omega }=C_{0}Au\omega \frac{\sin(3\omega t+\varphi )}{d}\;$$

where $C_{0}=\varepsilon_{0}\varepsilon bl/d$.

In a word, when AC voltage excitation was used, the 3*ω* signal was proportional to the amplitude *A*, exciting voltage *u*, and exciting frequency *ω*. The signal was also in the same phase as that of the vibrating amplitude of the resonator and can be taken as a detection parameter.

This detection system consists of a signal generator, a cancel link of the fundamental frequency signal, an amplifier, and a tracking band-pass filter. The output signals of the resonator include signals of the frequencies *f*_0_ and 3*f*_0_. Here, the signal of the frequency *f*_0_ was stronger than that of the frequency 3*f*_0_. Before the signal of the frequency *f*_0_ was enlarged, it had to be removed. *C*_1_ is the static capacitance of the resonator, and *C*_2_ is the capacitance of the reference capacitor. *U*_1_ is the voltage applied to the resonator, and *U*_2_ is the voltage applied to the reference capacitor. Here, *C*_1_ was 4 pF, and *C*_2_ was taken to be 5 pF. Thus, by adjusting the excitation voltage to obtain a relationship where *C*_1_*U*_1_ *= C*_2_*U*_2_, the fundamental frequency current in the resonator could be partially cancelled. A tracking band-pass filter was used to remove the frequency *f*_0_ current of the resonator completely.

The impedance of the signal generator was 50 Ω. To reduce the partial voltage on the signal generator, two voltage followers were used. An I/V converter and an amplifier were used to enlarge the weak current signals of nA by 10^−9^ A or 10^−12^ A orders of magnitude. 

With an I/V converter, the output signals of the mV order of magnitude can be obtained. The output signals should be further enlarged with an instrumentation amplifier and a differential amplifier. The fundamental frequency signal in the output voltage signals of the amplifier should be removed with the tracking band-pass filter, which consists of a programmable filter and a clock circuit. 

The frequency characteristics of the filters were only modulated by clock frequency. The center frequency was set to 1/50 of the clock frequency. The center frequency of the voltage signals was *f*_0_. This is then inputted into the programmable filter, and the hysteresis comparator changes the signals into square wave signals. The square wave signals are then inputted into the clock circuit and produces clock signals of 150 *f*_0_. The center frequency of the tracking band-pass filter is then locked to 3.4–44 kHz. 

**Figure 4:**
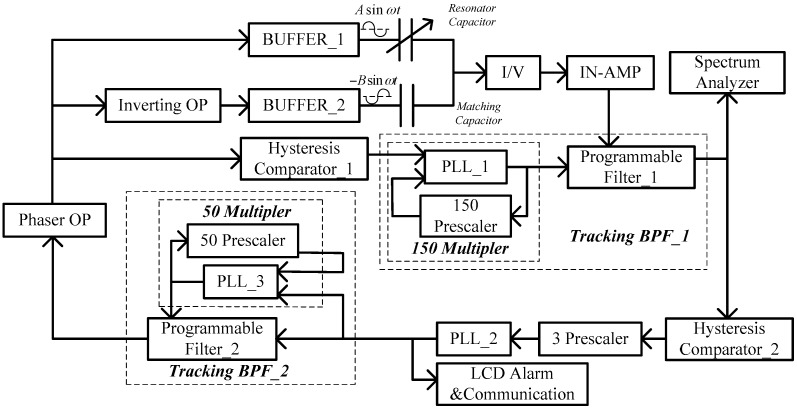
Block diagram of the self-exciting closed loop system.

Figure 4 shows a block diagram of the self-exciting closed loop system. *f_x_* denotes the frequency of the exciting signal and is similar to the first-order natural frequency *f*_0_ of the resonator. 

Output signals of the resonator and the reference capacitor include signals of the frequencies *f*_0_ and 3*f*_0_, which are applied to the I/V converter and the amplifier and then applied to the tracking band-pass filter. Its output signals are applied to the hysteresis comparator, a third frequency divider, and a phase-locked loop. Then, the signals are applied to another tracking band-pass filter. Through a phase shift amplifier, the signals are fed back to the resonator and the reference capacitor after they are fed to an inverting amplifier. Thus, the frequency *f_x_* of the exciting signal is locked to the first-order natural frequency *f*_0_ of the resonant beam. In addition, a positive feedback loop is used to increase the effective quality factor of the test system.

## 4. Experimental Results

**Figure 5:**
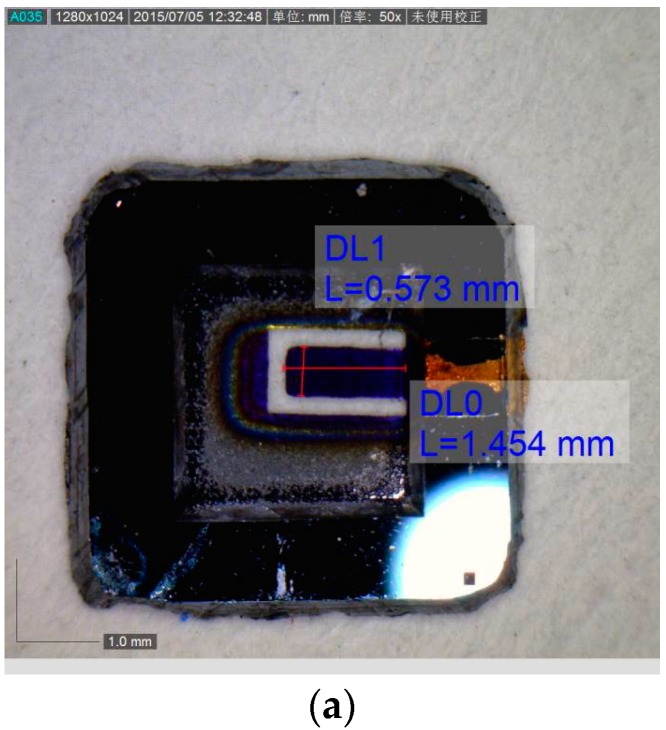

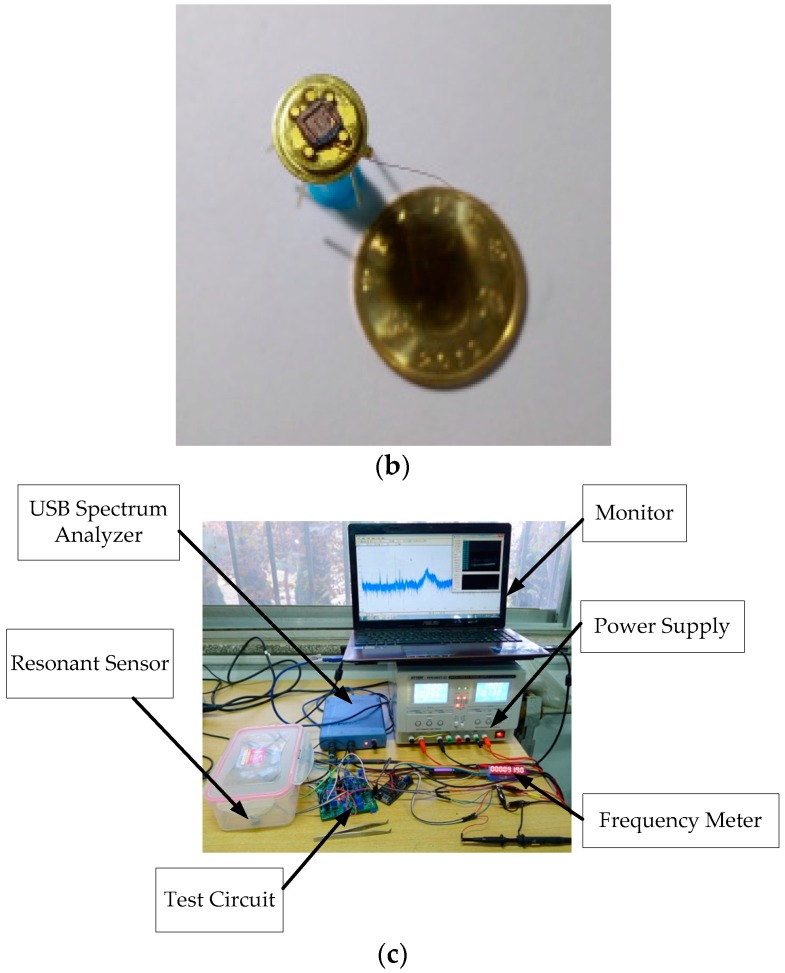
Micro-resonant gas sensor and its test system. (**a**) The cantilever; (**b**) the micro-resonant gas sensor; (**c**) the test system.

**Figure 6:**
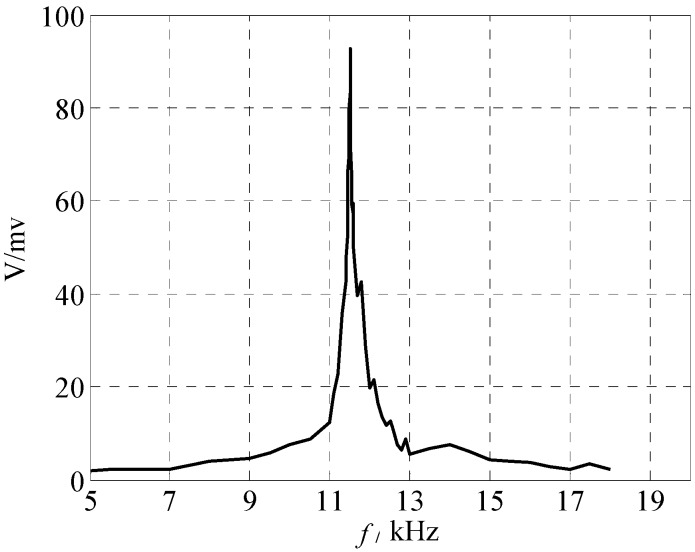
Open loop test results.

The resonant frequency and open-looped Q-factor of the micro-resonant gas sensor were measured with the above-mentioned measuring system. The micro-cantilever, the micro gas sensor, and its measuring system are shown in Figure 5. The open loop test results are given in Figure 6. The resonant frequency of the gas sensor was about 11.522 kHz in air and the quality factor Q was 144. A high stability of the measured resonant frequency was obtained (here, the mean square error is 0.066 Hz).

In order to determine the sensor’s sensitivity to ethanol vapor, the resonant frequency shift was measured when it was exposed to ethanol vapor. The concentration of ethanol was controlled by adjusting the quantity of ethanol vapor passing into an airtight container in which the sensor was placed. Here, an air valve was used to adjust the vapor quantity, and an ethanol concentration meter was used to monitor the vapor concentration.

**Figure 7:**
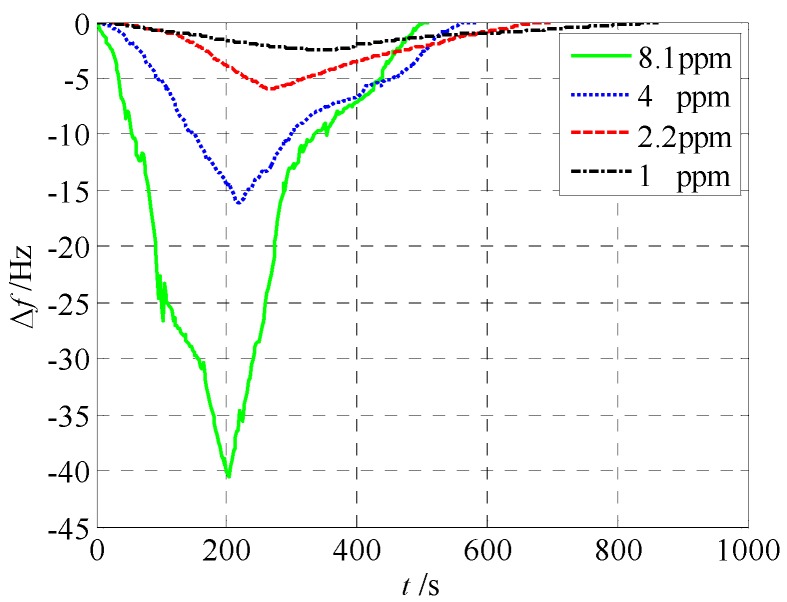
Real-time resonant frequencies for ethanol vapor.

Figure 7 gives the real-time resonant frequencies for an exposure of four different densities of the ethanol. The resonant frequency dropped by about 40 Hz when 8.1 ppm ethanol vapor was diffused into the phthalocyanine copper layer. At concentrations of 4 ppm, 2.2 ppm, and 1 ppm, the resonant frequency drops were 16 Hz, 6 Hz, and 2.5 Hz, respectively. Thus, the detection accuracy with respect to ethanol vapor using the sensor was about 0.4 ppm per Hz shift.

To make a comparison between the operating performances of the gas sensors with nanometer clearance and micrometer clearance, the capacitance between two poles, the resonant frequency, the open-looped Q-factor and the detection accuracy with respect to ethanol vapor of the micro-resonant gas sensor with 5 μm clearance were measured. The results are as follows.

For the sensor with 5 μm clearance, the capacitance between two poles was measured to be 2.2 pF and the capacitance between two poles for the sensor with 200 nm clearance was measured to be 39.6 pF. The capacitance difference between the two sensors of the different clearances was about 20-fold.

The resonant frequency of the gas sensor with 5 μm clearance was also about 11.522 kHz in air. The quality factor Q was 95 (about two-thirds of the quality factor Q for the sensor with 200 nm clearance), and the mean square error of the measured resonant frequency was 0.163 Hz (two times larger than the mean square error (0.066 Hz) for the sensor with 200 nm clearance). The detection accuracy with respect to ethanol vapor of the micro-resonant gas sensor with 5 μm clearance was about 25 ppm per Hz shift.

Thus, reducing the initial clearance between the pole plates to the nanometer level increases the capacitance between two poles such that the measuring accuracy of the micro-resonant gas sensor is increased by about 60 times.

**Figure 8:**
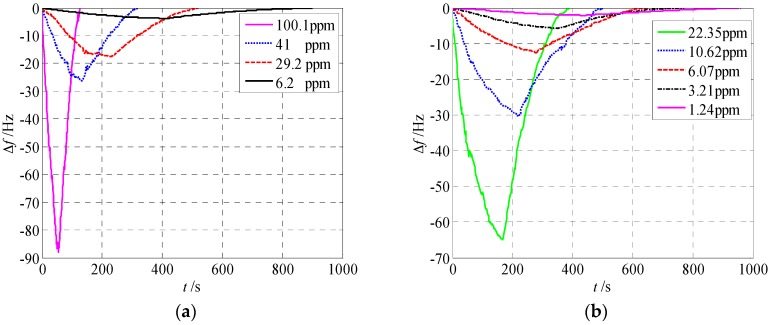
Real-time resonant frequencies for hydrogen gas and ammonia gas. (**a**) Hydrogen gas; (**b**) ammonia gas.

The gas sensor with nanometer clearance could be also used to monitor the concentration of other gases with good detection accuracy. Figure 8 shows the real-time resonant frequencies for exposure of different densities of hydrogen gas and ammonia gas. Results are as follows.

The resonant frequency dropped by about 85 Hz when 100.1 ppm hydrogen gas was diffused into the phthalocyanine copper layer. For 41 ppm, 29.2 ppm, and 6.2 ppm concentrations, the resonant frequency drops were 27 Hz, 18 Hz, and 3 Hz, respectively. The results show that the detection accuracy with respect to hydrogen gas using the sensor was about 3 ppm per Hz shift.

The resonant frequency dropped by about 65 Hz when 22.35 ppm ammonia gas was diffused into the phthalocyanine copper layer. For 10.62 ppm, 6.07 ppm, 3.21 ppm, and 1.24 ppm concentrations, the resonant frequency drops were 30 Hz, 12.5 Hz, 6.5 Hz, and 2.5 Hz, respectively. The detection accuracy with respect to ammonia gas using the sensor was about 0.5 ppm per Hz shift.

In a word, using the gas sensor with nanometer clearance, the detection accuracy (0.5 ppm per Hz shift) with respect to ammonia gas was similar to the detection accuracy (0.4 ppm per Hz shift) with respect to ethanol vapor. The detection accuracy was six times the detection accuracy with respect to hydrogen gas (3 ppm per Hz shift).

Results show that the proposed sensor has good detection accuracy for ethanol, hydrogen, and ammonia vapor and can be used to detect the density of any of the above-mentioned vapors. The sensor has no selectivity between ethanol, hydrogen, and ammonia vapor. If we want to distinguish the three vapors, we must measure the rates at which these vapors adsorb to the micro-sensor. From Figure 7 and Figure 8, we can find that the rate at which the ethanol adsorbs to the sensor is the fastest, and the rate at which the hydrogen adsorbs to the sensor is the slowest. For the detection of ethanol vapor, the best detection accuracy and the fastest measured speed can be obtained with the micro-sensor. Hence, phthalocyanine copper is suitable for detecting ethanol. Future work will focus on detecting mixtures of these three vapors. The rate at which the mixed vapor adsorbs to the sensor will be different from that of each of these three vapors individually.

## 5. Conclusions

In this paper, a fabricating process for micro-resonant gas sensors, by which the initial clearance between the pole plates of the gas sensors is reduced to 200 nm, is proposed. The resonant frequency shift of the sensor is measured when it is exposed to ethanol vapor, and the detection accuracy with respect to ethanol vapor was about 0.4 ppm per Hz shift. Reducing the initial clearance between the pole plates to the nanometer level increased the measuring accuracy of the micro-resonant gas sensor. The gas sensor was used to detect the gas concentration of hydrogen and ammonias, and good detection accuracy was obtained.
